# The influence of personality and ability on undergraduate teamwork and team performance

**DOI:** 10.1186/2193-1801-2-16

**Published:** 2013-01-19

**Authors:** Jinny Rhee, David Parent, Anuradha Basu

**Affiliations:** 1 San Jose State University, One Washington Square, San Jose, CA 95192-0087 USA; 2 San Jose State University, One Washington Square, San Jose, CA 95192-0084 USA; 3 San Jose State University, One Washington Square, San Jose, CA 95192-0070 USA

**Keywords:** Five-factor personality model, Big five personality model, Ability, Teamwork instruction, Capstone course

## Abstract

The ability to work effectively on a team is highly valued by employers, and collaboration among students can lead to intrinsic motivation, increased persistence, and greater transferability of skills. Moreover, innovation often arises from multidisciplinary teamwork. The influence of personality and ability on undergraduate teamwork and performance is not comprehensively understood. An investigation was undertaken to explore correlations between team outcomes, personality measures and ability in an undergraduate population. Team outcomes included various self-, peer- and instructor ratings of skills, performance, and experience. Personality measures and ability involved the Five-Factor Model personality traits and GPA. Personality, GPA, and teamwork survey data, as well as instructor evaluations were collected from upper division team project courses in engineering, business, political science, and industrial design at a large public university. Characteristics of a multidisciplinary student team project were briefly examined. Personality, in terms of extraversion scores, was positively correlated with instructors’ assessment of team performance in terms of oral and written presentation scores, which is consistent with prior research. Other correlations to instructor-, students’ self- and peer-ratings were revealed and merit further study. The findings in this study can be used to understand important influences on successful teamwork, teamwork instruction and intervention and to understand the design of effective curricula in this area moving forward.

**Electronic supplementary material:**

The online version of this article (doi:10.1186/2193-1801-2-16) contains supplementary material, which is available to authorized users.

## Background

Fostering effective teamwork in the curriculum is a necessity. The ability to work effectively on a team is highly valued by employers, in addition to communication and problem-solving skills ( [Thomas and Busby 2003]; National Academy of [Engineering 2004]). Students working as a team towards a common goal achieve more than if they work alone ( [Johnson and Johnson 1999]). Collaboration among students can lead to intrinsic motivation, increased persistence, and greater transferability of skills ( [Pfaff and Huddleston 2003]). Innovation is often sparked by teamwork involving the intersection of multiple disciplines ( [Haragon 2003]; [Denison and Kahn 1996]). Finally, teamwork is a learning outcome that is required for all engineering programs that are accredited by the Accreditation Board of Engineering and Technology programs (ABET).

Successful teamwork involves many intertwined factors. Many of us have observed teams of high ability individuals who never “gel” as a team, and consequently do not perform up to expectations. Similarly, there are teams of mediocre or even below-average players who somehow beat the odds and outperform more promising teams. Clearly, there are factors in successful teamwork beyond ability alone, and increased understanding of them has the potential for large impact in higher education, as well as in the workplace.

Personality traits are commonly studied as important individual-level factors in teamwork and team performance. There are many personality tests in existence, but a commonly accepted empirical model in the social sciences is called the Big-Five, or equivalently the Five-Factor Model (FFM) ( [Srivastava 2011]). The FFM describes a taxonomy of five personality domains which map traits that are correlated statistically. The five domains are: extraversion (outgoing, social), agreeableness (sympathetic, warm), conscientiousness (organized, dependable), emotional stability (calm, not easily upset), and openness (adventurous, creative). The FFM is based upon extensive, systematic, and rigorous empirical work, and is considered more viable as a model of personality than the well-known Myers-Briggs personality (Mc [Crae and John 1992]).

Team composition is commonly studied as an important group-level factor. Team composition can vary in gender, race, education, and functional background, in addition to measures of ability and personality.

Prior work has found correlations between individual personality and performance on a team. Neumann and Wright found that agreeableness and emotional stability predicted peer evaluations beyond skills and ability in a study of human resources teams ( [Neuman and Wright 1999]). In all fields, the degree of conscientiousness can be used to predict individual performance. Agreeableness was highly correlated to working successfully on teams. Extraversion and emotional stability positively influenced how a person felt about a work role (Ozer and Benet- [Martinez 2006]).

The personality composition of a team has been widely studied in a variety of disciplines and settings as predictors of team outcomes. A comprehensive compilation of group FFM clusters on engineering design team performance in the literature is presented in [Ogot and Okudan (2006]). Schilpzand et al. ( [2011]) found that graduate engineering and business student teams diverse in openness exhibited more creativity on their innovation management class project, as measured by existing creativity scales ( [Tierney and Farmer 2002]) and peer review. The hypothesis was that individuals high in openness promoted divergence and ideation while those low in openness promoted convergence and idea selection, both of which are necessary for team creativity. [Mohammed and Angell (2003]) found in a study of business student teams, that higher variability on agreeableness and emotional stability resulted in lower oral presentation scores, whereas higher variability on extraversion resulted in higher oral presentation scores. Neumann and Wright ( [Neuman and Wright 1999]) found that average measures of agreeableness and emotional stability at the team level also predicted supervisors’ ratings of team performance, accuracy, and work performed in their study of human resource teams, in addition to peer evaluations in the same study. In a study of manufacturing teams, the average extraversion and average conscientiousness of teams were both correlated with higher ratings from the supervisor for team performance (Barrick et al. [1998]). Peeters et al. (Peeters et al. [2006a]) found that an individual’s satisfaction with the team goes down if everyone on the team is extraverted, but these results seemed to be contradicted by another study published later by the same authors (Peeters et al. [2006b]). Shen et al. ( [2007]) found that there are some personality types that are better at the dual roles of engineering and design, but that a team should not be formed with more than one strong leadership type personality. It was also suggested that when forming teams to not let the students select their own teams, because it reduces the diversity required to have a successful team.

Some studies (Homan et al [2008]; Van Dick et al [2008]; Kearney et al. [2009]; Roberge and van [Dick 2010]) found that diversity in a team does not always increase a team’s performance, and as a result, diversity has to be managed carefully when selecting team members for a project. Peeters et al. (Peeters et al. [2006a]) also discovered that team members who rated themselves highly in conscientiousness felt dissatisfied with the team’s performance if the team had a high variance in conscientiousness. Team members, who were at a low level of conscientiousness, were not affected by those team members who were more conscientious (Peeters et al. [2006a]). The negativity generated by one person can also disrupt the performance of the entire team (Felps et al [2006]), regardless of the level of agreeableness of the other team members (Barrick et al. [1998]). Along the same lines, a dysfunctional team, with one or more members whose actions disturb the team, can result in members performing at a lower level than individuals working alone ( [Hsiung 2010]).

Interventions to improve teamwork and team performance based on personality considerations have been undertaken and studied in the prior literature. [Kapp (2009]) described the introduction of a team-building workshop into a senior capstone course in occupational safety and its subsequent beneficial effect in establishing collaborative environments conducive to learning. The workshop illuminated personality differences within the team, in addition to preferred work styles, expectations, and solutions for working together. Hutto et al. ( [2011]) formed teams using Fisher personality types (i.e. explorer, builder, negotiator, director) in a marketing class and found that it resulted in less conflict and more satisfaction with the experience than in self-selected teams. [Cunningham (2000]) presented a case study on the use of personality type in self reported success in managing an engineering undergraduate research group. Other case studies involving first time freshman engineering students reported the use of personality tests when communicating with other students ( [Whitman and Missingham 2009]; [Ogot and Okudan 2006]).

Ability within a group cannot be ignored when analyzing team performance. Steiner’s task typologies are commonly used to link measures of ability to team performance. The four typologies and hypothesized links to group ability are as follows: (1) additive tasks, where team performance is the sum of individual performance of team members, hypothesized to correlate to measures of mean ability of the group; (2) compensatory tasks, where team performance is proportional to the average of the individual contributions, also hypothesized to correlate to average group ability; (3) conjunctive tasks, where all team members must perform at some minimal level for successful team performance, hypothesized to correlate to the minimum ability member of the team; and (4) disjunctive tasks, where team performance is judged by best performance from any of the team members, hypothesized to correlate to the highest ability member of the team ( [Steiner 1972]).

There are many studies in the literature testing these hypotheses. Day et al. ( [2004]) argued that mean cognitive ability should predict all four types of tasks and finds some support for his hypothesis in a study of undergraduate psychology students, although mean, maximum, and minimum ability were all correlated. [Mohammed and Angell (2003]) found in their study of business students that higher mean cognitive ability of groups correlated with higher written report scores. Nihalani et al. ( [2010]) found that although class attendance and individual academic performance were positively correlated to group academic performance, groups with a superstar, or a group member with exceptionally higher performing member compared to the rest of the group, tended to score lower on group-level tasks. Their study was performed with teams comprised of psychology and statistics students. In a study of teams working in a manufacturing facility with tasks described as conjunctive, variability in cognitive ability within a team was positively correlated to both new ideas generated and team performance.

## Objectives and methodology

The overarching objective of the current study is to examine the influences of personality and ability on teamwork and team performance in the context of our multidisciplinary student population and culminating capstone projects in engineering and other disciplines. Personality and ability seem to be very important contributors to group dynamics, attitudes, and consequently performance, and worthy of targeted study in the current population. Increased understanding of these influences can be applied to designing and implementing more effective assignments and instruction in teamwork and team skills. Furthermore, this work, along with prior related literature, is another step towards understanding the pedagogy behind effective, wide-scale, multidisciplinary team-based instruction at all types of institutions, which is one of the eventual goals of this line of inquiry.

A distinction is often made between teams and groups in the literature. In this paper, we use both terms interchangeably to refer to tasks performed collectively by more than one person. The specific research questions addressed in this paper are the following:

- Question 1: How is group teamwork influenced by personality and/or ability?
- Question 2: How is individual performance on a team influenced by personality and/or ability?

Data was collected from five courses at a large public university. The courses were: mechanical engineering senior project, electrical engineering senior project, industrial design senior project, green entrepreneurship, and public policy. There were between 16 and 35 students in each course for a total of 121 undergraduate students, spanning four colleges at the university. Each course assigned a substantial group project comprising a large percentage of the overall course grade. The green entrepreneurship and public policy courses were one semester courses. The three senior project courses were two semester sequences; only data from the first semester is reported from these classes to maintain consistency with the other two courses. In all five courses examined, the scope of the group project assigned included research, analysis, and a proposal of a solution or action plan addressing a contemporary issue. These five particular disciplinary areas were chosen in this particular study because of their relevance to sustainable energy, a common theme in large culminating-type projects at our institution; we did happen to have one multidisciplinary project during the time period of this study involving subteams from all five participating courses. Nevertheless, this methodology can be applied to any collection of disciplines that might weigh in on other project themes. This study seeks to discover and understand trends that exist across disciplinary boundaries. It does not, therefore, investigate differences between the teams that were involved in the multidisciplinary project and those that were not.

The data collected for this study includes responses to online student surveys, and artifacts of student ability and achievement. At the start of the semester, a ten-item personality test developed by Gosling (Gosling et al. [2003]) was administered to all 121 students in all five participating courses. This instrument was reported to have a high degree of correlation with other instruments with significantly more items. At the end of the semester, a teamwork survey covering self and peer assessment was administered to all students in the participating courses. The teamwork survey used, shown in Additional file 1, generally rates engagement, leadership, and cooperation of team members, and was developed and tested by Van Duzer and Mc [Martin (2000]). Group scores on written reports and oral presentations were collected, where applicable. Student identification numbers were obtained, and students’ GPA, gender, major, and year in school were available to the study. Informed consent and confidentiality of the participants were implemented for this study, in compliance with the Institutional Review Board (IRB) at our institution. Statistical analysis was performed with IBM SPSS Statistics, Version 19. A standard t-test was used to judge if there were significant differences between the means from two groups. A one-way ANOVA procedure in conjunction with post-hoc tests were used to determine if there were any significant differences in means between more than two groups. Pearson’s correlation coefficient was computed to evaluate the strength of associations between dependent and independent variables. The probability-value, i.e. p-value, was used to judge statistical significance, with a p-value < 0.05 judged to be significantly small to rule out the null hypothesis (unless otherwise specified), as is conventionally interpreted.

Dependent variables describing team outputs included self-, peer-, and instructor-assessment of team skills, team performance and individual contributions. Team skills were quantified by students’ responses to survey questions. Team performance was quantified by students’ responses to survey questions and instructors’ scores on class deliverables. Individual contributions to the team were quantified using responses to the teamwork survey, both by the students themselves, as well as their teammates.

Independent variables affecting team outputs are often grouped into three categories: (1) individual-level factors, such as team member attributes, (2) group-level factors, such as team composition, and (3) environmental-level factors, such as task characteristics (Barrick et al. [1998]). In this study, independent variables included both individual-level and group-level characteristics described by FFM personality traits and ability. Ability was characterized by GPA in this study. Individual-level characteristics were the FFM personality scores and GPA for a given student. Group-level characteristics included the mean, maximum, minimum, and the difference between max and min for all FFM personality traits and GPA, for a given group. Environmental-level factors are not systematically examined in this study.

## Characteristics of students

Characteristics of the students in the participating courses were reported in a previous study (Rhee et al. [2010]) and are simply restated here as background information. The previous study focused on differences between a multidisciplinary project and disciplinary ones; the current study focuses on teamwork and team outcomes in particular. The student population was largely male-dominated as is typical in the participating disciplines, with the percentage male students in a class varying from 71% - 96%. The exception was the public policy class, which was 38% male. The engineering and industrial design courses are required of all seniors in their programs; hence we can infer that the percentages for these courses are fairly representative of those graduating in the discipline. The influence of gender bias in collaborative projects is outside the scope of this paper, and is simply noted for now.

The average GPAs of the courses participating in the study are listed in Table 1. They ranged from 2.56 to 3.12 with standard deviations ranging from 0.41 to 0.61. A series of post hoc tests revealed that the 0.56 difference in average GPA between the mechanical engineering senior project class and the green entrepreneurship class was statistically significant, with a p-value of 0.008. Otherwise, none of the other differences are statistically significant within the p-value threshold of 0.05.

**Table 1:** **Average GPA of courses participating in study**

	N	Average GPA	Std Dev.
Mechanical engineering senior project	24	3.12	0.41
Electrical engineering senior project	25	2.79	0.43
Industrial design senior project	17	2.97	0.44
Green entrepreneurship	16	2.56	0.61
Public policy	26	2.92	0.61

The average scores for each of the FFM personality attributes are shown in Table 2 for each of the five participating classes. The scale ranges from 1 to 7, with 7 indicating the maximum score for an attribute. In general, the differences reported between the disciplines are not statistically significant. Although it appears that that the business students are more extraverted than the rest of the group, and that industrial design students are more open to new experiences, a series of post hoc tests revealed that the differences in extraversion and openness have more than a 5% probability of the null hypothesis, indicating that they could be attributable to chance variations alone.

**Table 2:** **Average FFM personality scores for participating courses in study**

	N	Extraversio	Agreeable	Conscienc	Emotional	Openness
Mechanical engineering senior project	27	4.46	4.85	5.74	5.48	5.52
Electrical engineering senior project	26	4.21	5.12	5.87	5.19	5.48
Industrial design senior project	20	4.33	5.13	5.28	5.53	6.18
Green entrepreneurship	16	5.09	4.94	5.53	5.78	5.78
Public policy	32	4.63	4.52	5.67	5.12	5.55
Total	121	4.51	4.88	5.64	5.37	5.66

**Figure 1:**
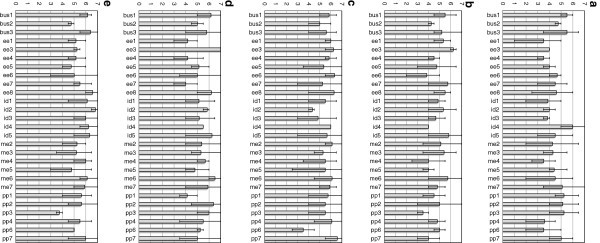
**(a) Average extraversion score for groups in current study, with error bars indicating maximum and minimum scores in the group. (b)** Average agreeableness score for groups in current study, with error bars indicating maximum and minimum scores in the group. **(c)** Average conscientiousness score for groups in current study, with error bars indicating maximum and minimum scores in the group. **(d)** Average emotional stability score for groups in current study, with error bars indicating maximum and minimum scores in the group. **(e)** Average openness score for groups in current study, with error bars indicating maximum and minimum scores in the group.

Student teams were identified in the five participating courses, and their average team personality attributes and GPA were computed. The teams were largely self-selected, except in a few instances where the interest in a particular project exceeded demand, in which cases there was some selection required by the instructor. In Figures 1(a) through 1(e), the team average personality attributes are graphed, and the maximum and minimum values in the group are indicated with error bars. The variability from group to group was quite large, and can be seen graphically in these figures. Teams with only one respondent were eliminated from the figures due to insufficient data.

**Figure 2:**
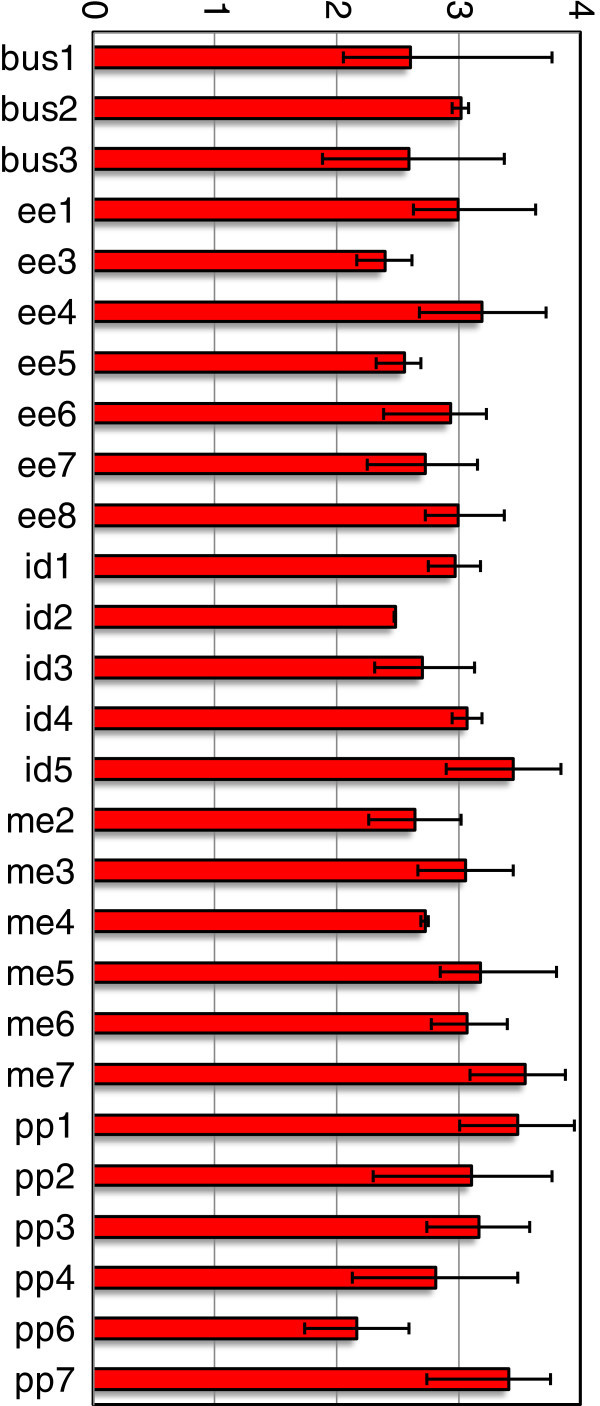
**Average GPA for groups in current study, with error bars indicating maximum and minimum GPAs in the group.**

The average GPA in each group is shown in Figure 2, again with the maximum and minimum values indicated with error bars. GPA is a measure of ability, and needs to be considered in the interpretation of the results. Generally speaking, the average GPAs of the groups are above 2.0, which is the minimum requirement for good standing in the undergraduate program. (There are, however, two groups with at least one member not in good academic standing, as shown on this figure). In addition, there do not appear to be any groups with a simultaneous low GPA average and a high GPA “superstar”, with the exception of possibly the “bus1” team. In other words, students with fairly similar GPAs appear to have chosen to work together for many of the groups in this study.

## Results and discussion

The research questions posed in this study are systematically examined in the context of our student population in this section.

### Question 1: how is group teamwork influenced by personality and/or ability?

For this analysis, the students’ rating of their groups’ sharing of responsibilities, resolution of conflict, and overall productivity were examined, along with the instructors’ assessment of group course deliverables. For the student rating, the students’ responses to the questions in Part I of the teamwork survey shown in Additional file 1 were used. For the instructor rating, the score on the group written report and oral presentation, if applicable, were used.

**Figure 3:**
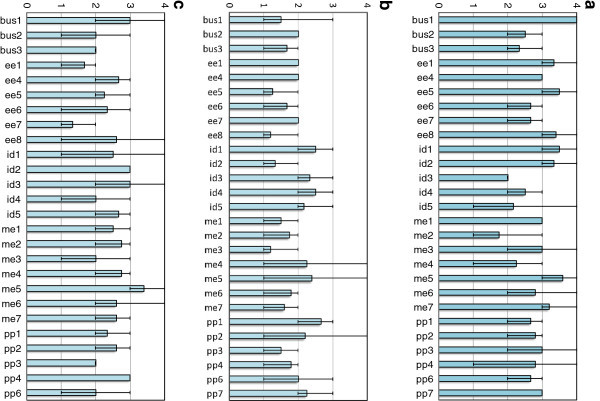
**(a) Average student response per group for the question, “To what degree did all members of the group share in the team’s responsibilities?” Possible responses were: (1) Some members did no work, (2) A few members did most of the work, (3) The work was generally shared by all members, and (4) Everyone did an equal share of the work.** Error bars indicate minimum and maximum responses in each group. For the entire sample, the average response was 2.90, and the standard deviation was 0.863. **(b)**. Average student response per group for the question, “Which of the following best describes the level of conflict at group meetings?” Possible responses were: (1) No conflict, (2) There were disagreements, but easily resolved, (3) Disagreements were resolved with considerable difficulty, and (4) Open warfare, still unresolved. Error bars indicate minimum and maximum responses in each group. For the entire sample, the average response was 1.83, and the standard deviation was 0.737. **(c)**. Average student response per group for the question, “How productive was the group overall?” Possible responses were: (1) Accomplished some, but not all of the project’s requirements (2) Met the project’s requirements but could have done much better, (3) Efficiently accomplished goals that we set for ourselves, and (4) Went way beyond what we had to do. Error bars indicate minimum and maximum responses in each group. For the entire sample, the average response was 2.44, and the standard deviation was 0.865.

The students’ responses to the questions in Part I of the team work survey are graphed in Figures 3(a)-(c), by group, with the minimum and maximum responses in each group indicated by error bars. Again, teams with only one respondent in the survey were not included in the plots. There was a fair amount of variation in responses from group to group both in mean and variability for all three questions as shown in the figures.

**Figure 4:**
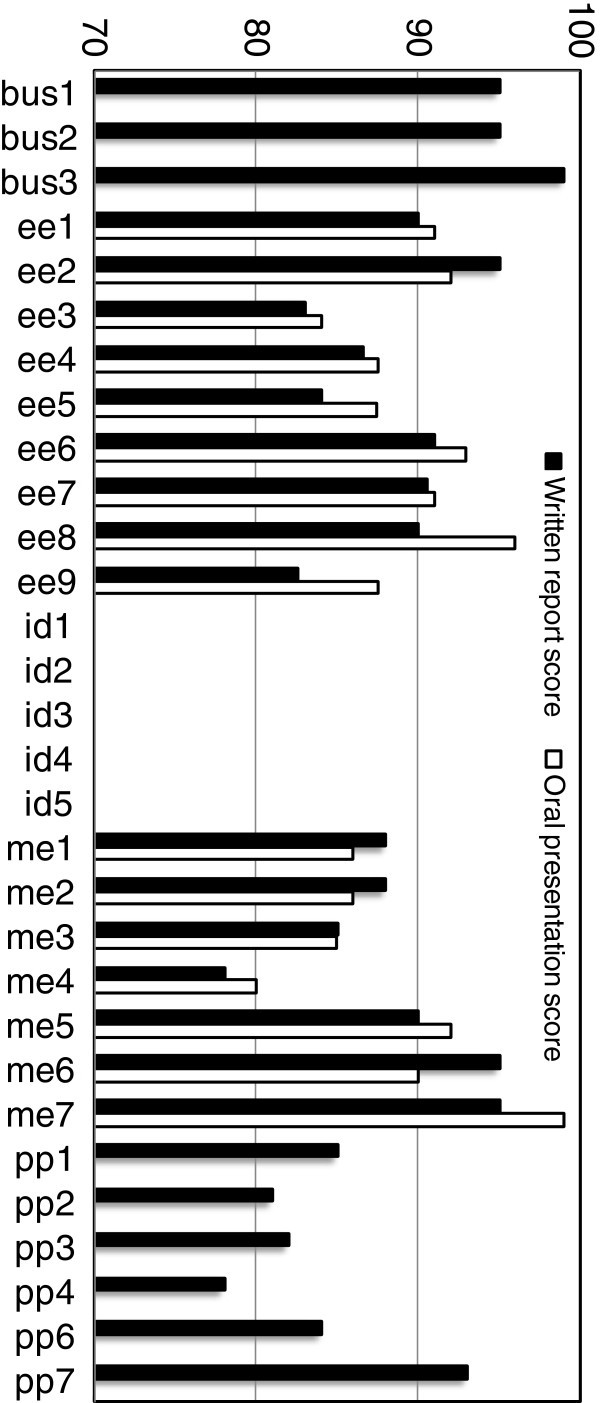
**Instructor rating of group written reports and oral presentations.** All courses, except for industrial design senior project, required a final written reportwith the same score given to all team members. The engineering senior project courses additionally required a final oral presentation with the same score given to all team members.

The scores (scaled to 100 points) received by each group by their instructor for their final written report and oral presentation, if applicable, are graphed in Figure 4. All courses, except for industrial design senior project, required a final written report with the same project score assigned to all team members. In the business (entrepreneurship) course, the score received by the students for grading purposes was a combination of the team score and peer evaluations; however, in this analysis, only the team score was used for consistency with the other courses. The engineering senior project courses additionally required a final oral presentation with the same score given to all team members. Although it was not possible to use the same grading rubric in each course, the scope and weight of the group assignments were similar in all courses in a disciplinary context, and if nothing else indicates the ability of the group to meet the requirements of the assignment.

As shown by Figures 3(a)-(c), the student groups report various degrees of success in achieving equal divisions of work, low conflict, and high productivity. Some groups exhibit complete agreement among members in response in some of the questions (e.g. see bus1, ee4, id3, me1, and pp7 in Figure 3a), while some groups exhibit significant variation (e.g. see id5 and pp4 in Figure 3a). These data were then probed to see if a student’s FFM traits and/or GPA were associated with their ratings by computing the Pearson’s correlation coefficients between them.

For the first question, “To what degree did all members of the group share in the team’s responsibilities?”, a student’s response was found to be negatively correlated to his or her emotional stability score on the personality survey (r = -0.229, p = 0.002). In other words, students who were more calm and less easily upset were less likely to feel that the group shared in the team’s responsibilities. This result was also reported in Rhee et al. ( [2010]), and is restated here for completeness of the current analysis. This result is contrary to the findings of previous studies (Ozer and Benet- [Martinez 2006]), which indicate that emotional stability is positively correlated to how a person feels about a work role. The explanation for the current result is unclear. Perhaps emotional stability in our student population is an indicator of apathy, and consequently describes less-engaged students. This result is statistically significant in our population and would be worth probing in a larger population involving other types of institutions, along with accompanying focus groups to obtain the required causal explanations.

For the second survey question, “Which of the following best describes the level of conflict at group meetings?”, a student’s response correlated positively with his or her GPA (r = 0.252, p = 0.012). In other words, a student with a higher GPA tended to report a higher level of conflict in his or her group in our population. A higher GPA student might be more perceptive and observant of group conflict than a lower GPA student, but again, this result bears further study.

There were no statistically significant correlations with FFM personality traits or GPA with the third survey question, “How productive was the group overall?” This result suggests that one student’s ability has little bearing on group performance perception.

The students’ responses to the three survey questions in Part I of the teamwork survey were then probed for correlations with team characteristics to determine if the composition of the group was associated with these outcomes. For each group, the mean, maximum, and minimum values for each of the FFM personality traits were computed, as well as the difference between the maximum and minimum values called the delta. The delta value was used to quantify the variability in a particular trait for a given group. All of these group characteristics were then used as independent variables.

The only statistically significant correlation from this exercise was a negative correlation between the second question (Which of the following best describes the level of conflict at group meetings?) and the mean agreeableness of the group (r = –0.215, p = 0.03). In other words, groups with a higher average agreeableness score tended to report less conflict. It is intuitive and not surprising that groups comprised of agreeable members generate and experience less conflict. There were no other significant associations with the other team characteristics, or for either of the other two questions in Part I of the teamwork survey.

What is noteworthy of this report is not only which correlations were significant, but also which ones were not. For example, there was no association found between students’ rating of group productivity and group GPA characteristics. Other correlations found in prior literature for self- and peer-rated team outcomes have not been replicated in this particular study.

Lastly, direct measures of team achievement were examined through the project deliverables in the courses studied. In all participating courses except for the industrial design senior project, a written report was required which was assigned a team grade. The same team grade was assigned to all team members. In the engineering senior design courses, an oral presentation was additionally required which assigned a team grade equivalently to all team members. Pearson’s correlation coefficients were computed for the scores in both cases, as shown in Figure 4, with the team composition attributes.

The written reports (and oral presentations, where applicable) were similar in scope in that they all required some research, analysis, and a proposed solution in each discipline. Due to the inherent variability in the topics covered, identical grading rubrics were not used for grading purposes, and reliability and validity measures were not computed. It is accurate to say, however, that the team grades in this study were measures of how well each group met the expectations of the instructor, which allows for some flexibility from discipline to discipline. The lack of validity and reliability between disciplines and instructors is noted as a limitation of this study, and should be considered in the interpretation of the results. For the data examined, extraversion was linked to both written report and oral presentation scores, and GPA was linked to oral presentation scores. The written report score was positively correlated to the mean extraversion score of the group (r = 0.533, p = 0.007) and the maximum extraversion score in the group (r = 0.465, p = 0.022). It was not correlated with the minimum extraversion or delta extraversion scores, nor was it correlated with any other FFM trait or any measures involving GPA. The oral presentation scores that were available were positively correlated to the maximum extraversion score in the group (r = 0.512, p = 0.043), the mean GPA of the group (r = 0.562, p = 0.024), and the maximum GPA score in the group (r = .524, p = 0.037).

Extraversion and cognitive ability of teams were found to be relevant in supervisor’s rating of team performance in a manufacturing environment in the prior literature (Barrick et al. [1998]). Extraversion is not generally correlated with individual performance in any field (Ozer and Benet- [Martinez 2006]), but is hypothesized to influence the social cohesion of a group which consequently affects group performance. In our study, groups with highly extraverted members and those with an exceptionally extraverted member tended to score highly on the written report. The extraverted members of the group appear to be able to compensate for a particularly introverted member if present, as evidenced by the lack of correlation to the minimum extraversion score. Furthermore, group attributes involving conscientiousness, which is a predictor of individual performance in all fields, were not a predictor of group written report performance, nor were attributes involving GPA, which are measures of group ability. In comparison, the oral presentation scores were correlated to maximum extraversion, as well as the mean and maximum GPA of the group. The correlation to maximum extraversion suggests that a highly extraverted group member positively impacts tasks involving oral presentation, and can compensate for more introverted members if they exist. The correlation to mean and maximum GPA suggests that team ability is an important predictor of an oral presentation task, and that high GPA individuals on a team can compensate somewhat for lower ones.

The association between oral presentation score with both mean and maximum GPA of the group suggests that a team’s oral presentation task has conjunctive and disjunctive characteristics. The group output is a sum of the individual contributions, and perhaps strong ability members shoulder the bulk of the responsibility. The written report task, on the other hand, did not exhibit correlation to team ability measures.

In summary, individual students’ perceptions that their group members shared equally in the responsibilities was negatively correlated to the individual’s emotional stability. The conflict reported by students was positively correlated to individual students’ GPAs, as well as negatively correlated to the mean agreeableness score of the group members. Written report scores were positively correlated to the mean and maximum extraversion scores in the group. Oral presentation scores were positively correlated to the maximum extraversion, mean GPA, and maximum GPA scores of the group.

### Question 2: how is individual performance on a team influenced by personality and/or ability?

For this analysis, individual performance on a team was quantified through student self-assessment and peer-assessment of their teammates. The instructor cannot be present for much of the interactions within a group, and the students are in a better position to rate the individual performance of his or her team members.

The Pearson’s correlation coefficient was computed between the students’ responses to the questions in Part II of the teamwork survey found in Additional file 1 and individual-level attributes. The questions on the survey cover performance aspects such as engagement, leadership, and accountability, and students were asked to rate themselves as well as each of their teammates. Self-assessment consisted of a student’s rating of himself or herself. Peer-assessment consisted of a student’s average scores from the rest of his or her team members.

Resulting statistically significant correlations between self-assessment and individual-level traits (i.e. GPA and FFM personality traits) are indicated in Table 3. Statistically insignificant correlations have been omitted for brevity. As shown by this table, there were a number of significant correlations with all of the independent variables except for openness. GPA is positively correlated with a student’s self-assessment of engagement, contribution of useful ideas, encouragement to be timely, and clear communication. Extraversion was negatively correlated to failing to do an equal share, and positively correlated to taking a leadership role, encouraging the group to be timely, and delivering promised work. Agreeableness was, unsurprisingly, negatively correlated to efforts to excessively dominate group discussions. Conscientiousness was positively correlated to being engaged, taking a leadership role, contributing useful ideas, encouraging group to be timely, delivering promised work, and clearly communicating. Emotional stability was positively correlated to contributing useful ideas and delivering promised work.

**Table 3:** **Statistically significant correlation coefficients between students’ self-assessment of individual performance on a team and individual-level traits (i.e. GPA, extraversion, agreeableness, conscientiousness, emotional stability, and openness)**

Aspects of Individual Performance	GPA	E	A	C	ES	O
Failed to do an equal share of the work.		r=-.214				
		p=.032				
Kept an open mind, was willing to consider others’ ideas.						
Was fully engaged in discussions during meetings.	r=.222			r=.217		
	p=.03			p=.032		
Took a leadership role in some aspects of the project.		r=.261		r=.279		
		p=.009			p=.005	
Often tried to excessively dominate group discussions.			r=-.228			
			p=.024			
Contributed useful ideas that helped the group succeed.	r=.316			r=.307	r=.219	
	p=.002			p=.002	p=.031	
Encouraged group to complete the project on a timely basis.	r=.311	r=.239		r=.267		
	p=.002	p=.018		p=.008		
Delivered work when promised/needed.		r=239		r=.478	r=.335	
		p=.018		p=0.00	p=.001	
Had difficulty negotiating issues with members of the group.						
Communicated ideas clearly and effectively.	r=.239			r=.285		
	p=.023			p=.008		

Resulting statistically significant correlations for peer-assessment are shown in Table 4. As shown by this table, the predictors of individual performance on a team based on peer-assessment exhibited quite a different pattern from those based on self-assessment. Most of the significant correlations in this exercise were with GPA, which correlated with peer-assessment of taking a leadership role, excessively dominating discussions, contributing useful ideas, encouraging group to be timely, delivering promised work, and clearly communicating. In addition, agreeableness was positively correlated with encouraging the group to be timely.

**Table 4:** **Statistically significant correlation coefficients between students’ averaged scores of individual performance from peer team members and individual-level traits (i.e. GPA, extraversion, agreeableness, conscientiousness, emotional stability, and openness)**

Aspects of Individual Performance	GPA	E	A	C	ES	O
Failed to do an equal share of the work.						
Kept an open mind, was willing to consider others’ ideas.						
Was fully engaged in discussions during meetings.						
Took a leadership role in some aspects of the project.	r=.307					
	p=.003					
Often tried to excessively dominate group discussions.	r=.209					
	p=.047					
Contributed useful ideas that helped the group succeed.	r=.316					
	p=.002					
Encouraged group to complete the project on a timely basis.	r=.297		r=.202			
	p=.004		p=.05			
Delivered work when promised/needed.	r=.327					
	p=.002					
Had difficulty negotiating issues with members of the group.						
Communicated ideas clearly and effectively.	r=.206					
	p=.05					

It is interesting to note that the attributes that are correlated with a student rating himself or herself favorably on individual performance were not necessarily the same as the attributes that appear to lead one’s peers to rate him or her favorably. Self-assessment of various aspects of individual performance on a team was correlated to GPA and all FFM personality traits except for openness, and arguably the most strongly with GPA, extraversion, and conscientiousness. Peer-assessment, on the other hand, was correlated most strongly with GPA.

An obvious question to ask is whether self-assessment correlates to peer-assessment. The correlation coefficient was computed for each question in Part II of the survey between a student’s self- and peer-rating for each semester. Significant results are summarized in Table 5.

**Table 5:** **Significant correlations between student self- and peer-assessment of teamwork**

Aspects of Individual Performance	Fall 2010 (N = 94)
Failed to do an equal share of the work.	r=0.255, p = 0.013
Kept an open mind, was willing to consider others’ ideas.	
Was fully engaged in discussions during meetings.	
Took a leadership role in some aspects of the project.	r=0.466, p=0.000
Often tried to excessively dominate group discussions.	r=0.300, p=0.004
Contributed useful ideas that helped the group succeed.	
Encouraged group to complete the project on a timely basis.	r=0.326, p=0.002
Delivered work when promised/needed.	r=0.306, p=.003
Had difficulty negotiating issues with members of the group.	r=.298, p= 0.004
Communicated ideas clearly and effectively.	

As shown by Table 5, there were moderate correlations between self- and peer-ratings for most of the questions rating individual performance on a team. However, it is noteworthy to point out that keeping an open mind, being fully engaged, contributing useful ideas, and clearly communicating, all showed no significant correlation between self- and peer-rating.

In addition, t-tests were performed using paired samples with the self- and peer-ratings in both semesters, and the following significant differences were found. Students rated themselves more favorably in taking leadership roles (Self: 3.27, Peer: 3.07, p = 0.021) and encouraging the group to be timely (Self: 3.45, Peer: 3.29, p = 0.04) compared to their peers’ rating of them. Although both questions showed positive correlation between self- and peer-assessment in Table 5, the differences between the two were statistically significant in our population.

In summary, self- and peer-assessments of individual performance on a team were largely, but not entirely, correlated, although self-assessment of individual performance exhibited correlation with a greater number of individual traits. There was a tendency for students to rate themselves more favorably than their peers in some of the individual performance questions posed.

### Multidisciplinary project – a brief examination

Research on effective teamwork in multidisciplinary teams is increasingly important as universities strive to incorporate it into curricula. In the current study, one student group from each of the five participating courses in this study also collaborated on a multidisciplinary project of mutual interest – in this case, the design and construction of a solar-powered house. Each student group was graded for their disciplinary piece of the overall project by their course instructor, but was required to exchange information and collaborate with the other teams outside of their discipline to do so. There are, of course, other significant outcomes required for successful multidisciplinary projects compared to disciplinary ones, such as the need to identify contributions, information needs, and constraints of multiple fields, as well as valuing, integrating, and learning from multiple fields (Paretti et al. [2010]). This is outside the scope of this particular paper, however, and the examination here will be limited to a preliminary examination of teamwork in a multidisciplinary context.

Although the sample size is small (only 5 participating teams), this pilot effort was briefly examined in the context of the current teamwork analysis. The students participating in the multidisciplinary project were asked to rate each of the participating disciplinary teams using the questions in Part II of the survey. Students’ ratings of their own team were averaged for each question and denoted the ‘self team’ rating. A team’s ratings from students in the remaining four disciplinary teams were averaged for each question and denoted the ‘peer team’ rating.

Unlike the disciplinary teams, the self team- and peer team-ratings were not well-correlated in the multidisciplinary project case study. The only significant correlation between self- and peer-ratings was “difficulty negotiating” (r=0.929, p=0.020, N=5). Furthermore, peer team-rating was generally more negative than self team-rating. Significant differences indicated by a t-test included the items “delivered work when promised” (Self: 3.71, Peer: 3.07, p = 0.017) and “communicated ideas clearly and effectively (Self: 3.57, Peer: 3.04, p = 0.049).

This preliminary analysis highlights the increased importance of effective communication in multidisciplinary projects. It is likely that additional skills beyond those needed in disciplinary projects are required. The importance of negotiation skills is shown by the high awareness of teams having trouble negotiating, as indicated by the strong correlation between the self team- and peer team-ratings. In addition, the significant discrepancies between the self team- and peer team-ratings suggest a tendency to value one’s own disciplinary contributions over others and/or difficulty communicating disciplinary contributions to others.

### Limitations of study

There are several important limitations to note about this study. First, the sample was drawn from five specific courses at our large public university, and was male-dominated. Although this is typical of the disciplines involved in this study, to what extent this sample is representative of the upperclassmen population in higher education as a whole is not known. Reliability and validity measures were not computed in the written report and oral presentation grades between instructors due to the inherent differences in disciplinary topics covered, and should be considered in the interpretation of the results. Also, some of the courses in the current study were the first semester course in a two-course sequence. The student teams in this study were formed at the start of the semester and were studied for one semester. Student teams in formation for a longer period of time may exhibit different dependencies and dynamics as the members get to know each other than newly formed teams. Finally, the scope of the project assigned to the groups was not varied in this study, and the results may change if the nature of the task assigned is also changed.

## Conclusions

The purpose of this study was to examine the influences of personality and ability on teamwork, team performance, and individual performance on a team, in a multidisciplinary student sample. It measured personality using the five-factor model personality traits and measured students’ ability in terms of GPA. The dependent variables included self-, peer-, and instructor-assessment of team skills, team performance and individual contributions.

Notwithstanding its limitations discussed in the previous section, our study presents several findings, which are summarized below:

·Ability and personality on group performance: Students’ perception that their group members shared equally in the responsibilities was negatively correlated to their emotional stability. This result is contrary to that of prior studies (Ozer and Benet- [Martinez 2006]) and could mean that emotional stability in our student sample indicates apathy, and consequently, less-engaged students. The conflict reported by students was positively correlated to his or her GPA, as well as unsurprisingly negatively correlated to the mean agreeableness score of the group members. Written report scores were positively correlated to the mean and maximum extraversion scores in the group. Oral presentation scores were positively correlated to the maximum extraversion, mean GPA, and maximum GPA scores of the group. The correlation between extraversion and written report/oral presentation scores is consistent with prior research (Barrick et al. [1998]). The correlation between ability and oral presentation scores is also consistent with the findings of previous studies ( [Mohammed and Angell 2003]). These results merit further investigation in a larger and gender-diverse population.

On the other hand, ability measured by GPA scores was negatively related to students’ assessment of teamwork in terms of the degree of conflict within the team. This implies that ability could be related both positively and negatively to team performance depending on the aspect of performance being measured and whose assessment we look at, whether that of team members (students) or external assessors (instructors).

Implications for instructors and teams can be deduced from these results. Understanding the positive roles of extraversion in team composition in addition to ability can help form or guide effective teams. Along the same lines, high emotional stability or ability has been shown to have negative impacts on team perception, and this awareness can be used to address related issues if they arise.

·Ability and personality on individual performance on team: Individual performance was measured by ten questions that focused on aspects such as engagement, leadership, and communication. Self- and peer-assessments of individual performance on a team were largely, but not entirely, correlated. Self-assessment of individual performance exhibited correlation with GPA and all of the FFM personality traits except openness. Peer-assessment largely correlated with GPA. There was a tendency for students to rate themselves more favorably than their peers on (a) taking a leadership role and (b) encouraging the group to be timely.

Implications for instructors and teams, again, include promotion of increased awareness of trends found and application of the results. Furthermore, the role of GPA and the FFM personality traits on self- and peer-assessment, in addition to the tendency of students to rate themselves more favorably than their peers can be used in the interpretation of group conflict descriptions, and ultimately the course of action if teaching moments or intervention is required.

Furthermore, a pilot multidisciplinary project team consisting of five disciplinary teams of approximately five members each was briefly examined in the context of this study. There was agreement as to which disciplinary teams were having difficulty negotiating. In addition, the disciplinary teams tended to rate themselves much more favorably than the peers outside of their discipline in (a) delivering work when promised and (b) clearly communicating. These results suggest the need for better negotiation and communication skills to manage diversity across disciplinary teams to promote a better understanding of different disciplinary backgrounds, cultures, and contributions.

The findings in this study illuminate associations between personality, ability, and teamwork for a type of task that is commonly assigned as a group project at many universities. The range of correlations found in this study and their comparison to the prior literature indicates the dependence of the results on the population and/or the environmental-level factors that vary from study to study. This dependence is not well-understood, and merits further research. This study outlines the results found in our multidisciplinary undergraduate population and has possible extension to other similar populations. It is the hope that by better understanding important influences on successful teamwork, teamwork instruction and intervention will continue to become more effective.

## Recommendations for future work

This work answered some questions, and raised many others. Suggestions for instructors seeking to apply the results of this and related papers, or for education researchers continuing this line of inquiry include the following:

- Instructional materials promoting awareness of personality influences on teamwork could be developed for project-based team instruction. The awareness itself may help students predict, explain, and help resolve difficulties that often arise in teams. For example, extraverted individuals on a team appear to be correlated with various measures of team performance. Teams without extraverted members who are aware of the correlation could try to compensate in other ways such as development of other social skills.
- Effective intervention strategies for instructors supervising group projects could be developed. Instructors (or managers) seeking to form high-performing teams, or those seeking to teach students how to maximize productivity in any given team would benefit from such strategies.
- Further research is required to map correlations that exist for external-level factors (e,g. type of institution, type of task assigned, reward structure, etc.).
- Standardized rubrics for quantifying achievement on written reports and oral presentations could be developed and used to ensure validity. A calibration routine among instructors using the rubric could also be developed to ensure reliability.
- Lastly, the influence of personality and ability in project teams that span more than one semester was not studied in this work, and would be of interest in longer term projects.

## Authors’ information

Jinny Rhee is an Associate Professor in the Department of Mechanical and Aerospace Engineering at San JoseÂ´ State University, where she has been since 2002. Her interests include renewable energy technologies, thermal management of electronics, and multidisciplinary and interdisciplinary education for engineers. Dr. Rhee completed a B.S. (1989), M.S. (1990), and Ph.D. (1995) at Stanford University, all in mechanical engineering. Dr. Rhee is an active member of ASME, IEEE—CPMT (Components, Packaging, and Manufacturing Technologies), and SWE (Society of Women Engineers).

David Parent is an Professor in the Department of Electrical Engineering at San JoseÂ´ State University where he has been since 1999. His passion is bringing practical engineering experiences to the classroom and using his research in the multidisciplinary areas of solar cell fabrication, artificial neurons, and neural interfacing as case studies for undergraduate and masters degree students. He earned his BSEE (1992), MSEE (1996) and Ph.D (1999) from the university of Connecticut. He is an active member of IEEE.

Anuradha Basu is a professor in the Department of Organization and Management in the College of Business, and Director of the Silicon Valley Center for Entrepreneurship, at San Jose State University. Her research interests include the impact of entrepreneurship education and immigrant entrepreneurship. She obtained a BA (Honors) degree in Economics from the University of Delhi, India, an MBA from the Indian Institute of Management, Calcutta, and MPhil and PhD degrees in Economics from the University of Cambridge, UK.

## Electronic supplementary material

Additional file 1: **Online survey questions on teamwork.** (DOC 50 KB)
