# Supplementary material for: The influence of personality and ability on undergraduate teamwork and team performance
Source: Springerplus. 2013 Jan 19;2:16. doi: 10.1186/2193-1801-2-16 (PMC3568485; doi:10.1186/2193-1801-2-16)
Supplement: Supplementary file 1 — Additional file 1: Online survey questions on teamwork. (DOC 50 KB) [file 40064_2012_68_MOESM1_ESM.doc]

Additional file 1

Online survey questions on teamwork

Part I: Students’ assessment of teamwork

To what degree did all members of the group share in the team's responsibilities?

(1)Some members did no work at all.

(2)A few members did most of the work.

(3)The work was generally shared by all members.

(4)Everyone did an equal share of the work.

Which of the following best describes the level of conflict at group meetings?

(1)No conflict, everyone seemed to agree on what to do.

(2)There were disagreements, but they were easily resolved.

(3)Disagreements were resolved with considerable difficulty.

(4)Open warfare: still unresolved.

How productive was the group overall?

(1)Accomplished some but not all of the project's requirements.

(2)Met the project requirements, but could have done much better.

(3)Efficiently accomplished goals that we set for ourselves.

(4)Went way beyond what we had to do, exceeding even our own goals.

Part II: Students’ self- and peer-assessment of individual performance on team

Please rate yourself and each of your teammates in the following areas:

|  | 1. Disagree | 1. Tend to Disagree | 1. Tend to Agree | 1. Agree |
| --- | --- | --- | --- | --- |
| Failed to do an equal share of the work. |  |  |  |  |
| Kept an open mind, was willing to consider others’ ideas. |  |  |  |  |
| Was fully engaged in discussions during meetings. |  |  |  |  |
| Took a leadership role in some aspects of the project. |  |  |  |  |
| Often tried to excessively dominate group discussions. |  |  |  |  |
| Contributed useful ideas that helped the group succeed. |  |  |  |  |
| Encouraged group to complete the project on a timely basis. |  |  |  |  |
| Delivered work when promised/needed. |  |  |  |  |
| Had difficulty negotiating issues with members of the group. |  |  |  |  |
| Communicated ideas clearly and effectively. |  |  |  |  |
